# Removal at birth and its challenges for midwifery care

**DOI:** 10.18332/ejm/146647

**Published:** 2022-04-05

**Authors:** Kaat De Backer, Kate Chivers, Claire Mason, Jane Sandall, Abigail Easter

**Affiliations:** 1 Department of Women and Children's Health, School of Life Course and Population Sciences, Faculty of Life Sciences & Medicine, King's College London, London, United Kingdom; 2 Birth Companions, London, United Kingdom; 3 Centre for Child and Family Justice Research, Lancaster University, Lancaster, United Kingdom

**Keywords:** child protection, maternity care, safeguarding, loss of custody

## Abstract

In England, care proceedings refer to the process whereby the family court decides to remove a child from its parents against their wish, due to a heightened risk of significant harm. There has been a worrying increase of the number of babies that are removed shortly after birth due to care proceedings in England. The removal of a newborn baby from its parents often occurs while the mother is still recovering in hospital and is a deeply distressing, intrusive and emotionally impactful event, both for parents as well as for midwives involved in their care.

Although the number of removals of newborn has risen, increasing support for those involved has not followed the same pace. These women are particularly vulnerable after the removal of a child but there is a lack of evidence and guidance to improve the experiences and the perinatal outcomes of these mothers and their infants. At a healthcare professional level, the impact of care proceedings and removals at birth on the midwifery workforce cannot be underestimated and has been described as one of the most challenging aspects of contemporary midwifery practice. In order to improve the care and outcomes of this under-researched and often stigmatized group of mothers, midwives need to have access to adequate training and supervision. Against the current challenges within UK maternity services, this is of the utmost importance to prevent further burnout among midwives.

## INTRODUCTION

In England, the protection of the welfare of children is laid out in the Children Act of 1989. Healthcare professionals working with pregnant women, including midwives, have a duty to refer the unborn baby to Children’s Social Care, when safeguarding concerns have been identified in pregnancy. In the most worrying cases, local authorities may apply to the family courts for an ‘Interim Care Order’ (ICO) once the baby has been born, which may lead to removal of the baby if the threshold (risk of significant harm attributable to the parents’ care or omission of care) is sufficiently met and proven. Babies, placed in State Care in the first week of life, have been described as being ‘born into care’^1,2^.

Recent reports have raised concerns about the number of babies in State Care within a week after birth (2914 babies in 2019–2020), the rapid increase within the last decade (from 26 per 10000 live births in 2007–2008 to 48 per 10000 live births in 2017–2018, a 142% increase), and the regional differences, with the highest figures reported for the North of England, where one baby in every 46 live births was ‘born into care’^1,2^. A complex set of reasons, such as austerity, family poverty and availability of residential mother and baby foster placements, has been highlighted as contributing factors to these wide regional variations^3^.

## COMMENTARY

The separation of a newborn baby from its birth parents often occurs while the mother is still recovering in the maternity hospital and is a deeply distressing, intrusive and emotionally impactful event, both for birth parents as well as for professionals involved in their care. A recent independent review of Children’s Social Care in the UK acknowledged that more needs to be done to support parents who have their child removed from their care^4^. They often experienced childhoods marked by adversity and abuse, and almost half were in care themselves^5^. Recent studies found that women in care proceedings have higher rates of mental health needs prior to and during pregnancy when compared to matched comparison groups^6,7^. In addition, the 2021 Confidential Enquiry into Maternal Deaths in the UK highlighted a large proportion of women who died by suicide or from substance misuse were known to social services (37% and 66%, respectively) and had their baby taken into care (16% and 43%, respectively)^8^. In the immediate postnatal period, mothers whose baby is taken into care face an acute psychosocial crisis, which can trigger a return to harmful coping strategies, such as misuse of drugs and alcohol^9,10^. One third of women will return to the courts in a very short period (mean interval 17 months), often after the birth of another baby. This sequence of rapid repeat pregnancies carries significant health risks for both mother and baby and compounds previous trauma and loss^11^.

Engagement with antenatal services is crucial and will inform the decision of Children’s Social Care to apply for an Interim Care Order. However, women have reported to feel overwhelmed by the number of professional agencies involved in their care without clear oversight or joint working^12^, and described feelings of self-judgement as a ‘bad mother’, alongside facing social stigma and judgement by professionals^13^. This could lead to non-engagement, exacerbated by previous poor experiences of services’ involvement^14^. In addition, access to specialist support, including perinatal mental health services, is often not available to these women. In combination with a significant reduction of postnatal midwifery follow-up in recent years in the UK (not in the least since the COVID-19 pandemic), and lack of designated professional support following removal of the baby, these women fall between the gaps and are left with limited or no support. Local and national charities and services across the UK, are filling this void but are facing a daunting and increasingly overwhelming task.

In order to improve the care and outcomes of this under-researched and often stigmatized group of mothers, more research is required into the maternity care experiences of birth mothers and those who support them, the role of midwives during this challenging time and the impact this has on the midwifery workforce. Continuity of care has been found to be beneficial for women with social risk factors^15^ and implementation of trauma-informed care in maternity services will contribute to a reduction of stigma and shame and re-traumatization of already vulnerable mothers^16^. Initiatives where integrated, compassionate and multidisciplinary care is offered to parents who face removal of their children, such as the alternative court model of the Family Drug and Alcohol Courts (FDAC) in England, have shown promising results, with evidence suggesting higher substance misuse cessation and family reunification rates for FDAC participants compared to cases heard in ordinary courts^17^.

Midwives are trained to provide needs-based holistic care throughout pregnancy, birth and the postnatal period and can play a vital role in supporting these women. However, the impact of care proceedings and removals at birth on the midwifery workforce cannot be underestimated and has been described as one of the most challenging aspects of contemporary midwifery practice. Midwives in the UK are bound by the Code of the Nursing and Midwifery Council (https://www.nmc.org.uk/standards/code/), which sets out the professional standards of practice and behavior. The Code stipulates midwives have to act ‘in the best interest of people at all times’ but lacks guidance how to do so in the case of conflicting interests in the context of care proceedings. Mason et al.^18^ recently issued the first set of best practice guidelines to address these lacunae in care. These include the provision of a specialist pathway of midwifery care for women at risk of separation at birth and specialist training for midwives in trauma-informed care, to help midwives consider the needs of women in this situation. This is most welcome, as very rarely do midwives receive any training pre- or post-registration training on how to navigate this emotional and professional complex dilemma^19^.

The conflicting roles of midwives in this matter, being the advocate and care-provider of the woman on one hand, and the safeguard of the (unborn) baby on the other, creates competing values, and contradicts core midwifery values of respect for women, informed choice and consent and a desire to provide woman-centered care. Festinger’s cognitive dissonance theory has been used in this context as it suggests we attempt to hold our attitudes and beliefs in harmony and avoid disharmony (or dissonance)^20^. When midwives are unable to reconcile their actions with the beliefs and values they are so passionate about, they experience moral distress, which can manifest in anger, sadness or anxiety^21^. Long-term professional impact of moral distress has been linked with midwives withdrawing from caring and becoming less sensitive to the needs of women in their care and/or choosing to work in casual and agency-based employment^22^. As such, it is a matter of workforce retention to put evidence-based support structures in place to alleviate this burden on the midwifery workforce.

## CONCLUSION

Concerns about midwives’ mental health and well-being and its impact on workforce retention have never been more prominent. Two years of COVID-19 pandemic challenges have left many maternity services in the UK struggling with staff shortages. Maternity staff, who were already facing a challenging job before the pandemic, are reporting to feel burned out, with many leaving their job or the profession altogether. The Royal College of Midwives warned of a ‘midwife exodus’ as it published results of a recent survey among midwives: over half of the midwives surveyed said they were considering leaving their job as a midwife, with 57% saying they would leave the NHS in the next year^23^. These alarming figures are a disastrous forecast, for the entire midwifery profession, and for women and babies within their care. But it will be even more detrimental to those women and babies that are in desperate need for compassionate and integrated midwifery care, as they will – once more – fall between the gaps.

## Data Availability

Data sharing is not applicable to this article as no new data were created.
